# A crucial role of neutrophil extracellular traps in pulmonary infectious diseases

**DOI:** 10.1016/j.pccm.2023.10.004

**Published:** 2024-02-03

**Authors:** Ting Pan, Jae Woo Lee

**Affiliations:** a Shanghai Key Laboratory of Lung Inflammation and Injury, Department of Pulmonary Medicine, Zhongshan Hospital, Fudan University, Shanghai 200032, China; b Department of Anesthesiology, University of California Los Angeles, Los Angeles, CA 90230, USA

**Keywords:** Pulmonary infectious diseases, Neutrophils, Neutrophil extracellular traps (NETs), NETosis

## Abstract

Neutrophil extracellular traps (NETs), extrusions of intracellular DNA with attached granular material that exert an antibacterial effect through entangling, isolating, and immobilizing microorganisms, have been extensively studied in recent decades. The primary role of NETs is to entrap and facilitate the killing of bacteria, fungi, viruses, and parasites, preventing bacterial and fungal dissemination. NET formation has been described in many pulmonary diseases, including both infectious and non-infectious. NETs are considered a double-edged sword. As innate immune cells, neutrophils release NETs to kill pathogens and remove cellular debris. However, the deleterious effects of excessive NET release in lung disease are particularly important because NETs and by-products of NETosis can directly induce epithelial and endothelial cell death while simultaneously inducing inflammatory cytokine secretion and immune-mediated thrombosis. Thus, NET formation must be tightly regulated to preserve the anti-microbial capability of NETs while minimizing damage to the host. In this review, we summarized the recent updates on the mechanism of NETs formation and pathophysiology associated with excessive NETs, aiming to provide insights for research and treatment of pulmonary infectious diseases.

## Introduction

Neutrophils are a part of the human innate immune system.^1^ The primary role of neutrophil is defense against microorganism invasion through phagocytosis, the release of reactive oxygen species (ROS), and degranulation.^2^ In addition, neutrophil extracellular traps (NETs), which are extrusions of intracellular DNA and the attached granular material that exert an antibacterial effect through entangling, isolating, and immobilizing microorganisms, have been extensively studied in recent decades.^3^^,^^4^ NETs are composed of extracellular DNA (eDNA) fibres coated with antimicrobial proteins, including histones, neutrophil elastase (NE), myeloperoxidase (MPO), and α-defensins.^5^

The primary function of NETs is to entrap and facilitate the killing of bacteria, fungi, viruses, and parasites, preventing bacterial and fungal dissemination.^6^, ^7^, ^8^ Nevertheless, these attributes make NETs potentially detrimental to the host. An increasing body of evidence suggests that NETs have a direct cytotoxic effect on lung epithelial and endothelial cells. Excessive NET production has been associated with the occurrence and exacerbation of many lung diseases, which can lead to a vicious cycle of recruitment-inflammation-recruitment responses.^9^^,^^10^ In addition, the thick sticky secretions produced by NETs containing eDNA and proteins not only tend to cause airway obstruction but also provide a niche to establish the infection, promoting bacterial colonization and biofilm formation.^11^, ^12^, ^13^, ^14^ Clinical and animal research has demonstrated the benefits of targeted inhibition of NETs in alleviating lung injury and thrombosis.

For pulmonary infectious diseases, in particular, clinical outcomes depend on the balance between the inflammatory response and pathogen clearance. Thus, maintaining a critical balance of NETs may be a potential therapeutic target. In this review, we summarize recent studies on the pathophysiological role of NETs in pulmonary infectious diseases, as well as some experimental and clinical methods to modulate their harmful effects.

## The formation of NETs

**Fig. 1:**
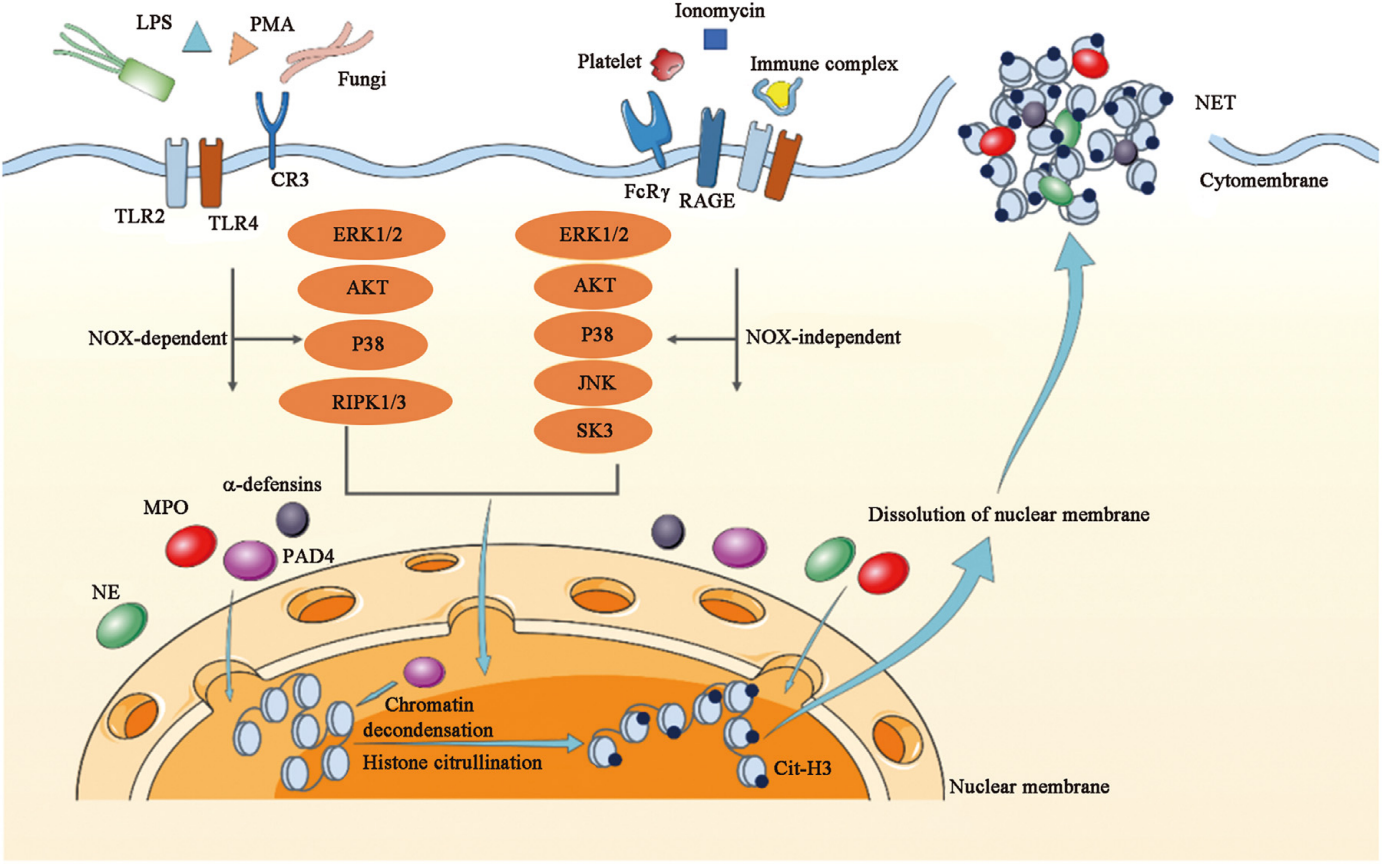
The formation of NETs. Various stimuli induce either NOX-dependent or NOX-independent NET formation with the activation of different sets of kinases. These processes enable enzymes such as NE, MPO, and PAD4 to enter the nucleus and decorate the chromatin. Finally, the nucleus disintegrates and NETs are released. AKT: Protein kinase B; Cit-H3: Citrullinated histone H3; CR3: Complement receptor 3; ERK: Extracellular regulated protein kinase; FcRγ: Fc gamma receptor; JNK: c-Jun N-terminal kinase; LPS: Lipopolysaccharide; MPO: Myeloperoxidase; NE: Neutrophil elastase; NETs: Neutrophil extracellular traps; NOX: nicotinamide adenine dinucleotide phosphate (NADPH)-oxidase; PAD4: Protein arginine deiminase 4; PMA: Phorbol 12-myristate 13-acetate; RAGE: Receptor for advanced glycation endproducts; RIPK: Receptor-interacting protein kinase; SK3: Small conductance calcium-activated potassium channel protein 3; TLR: Toll-like receptor.

It is increasingly recognized that neutrophils have additonall model of death aside from apoptosis and necrosis.^15^ Most current studies have shown that most neutrophils release NETs while dying 2–4 h after their activation,^16^ and this process has been called NETosis. As a novel antimicrobial defense system, NET formation is induced by various microorganisms and pro-inflammatory mediators, including those by potent activator phorbol 12-myristate 13-acetate (PMA), lipopolysaccharide (LPS), bacteria, viruses, cigarette smoke, and some environmental factors. NETosis can also be induced by cytokines, chemokines, immune complexes and other physiological stimuli.^17^^,^^18^ The various different stimuli induce ROS formation which can activate different sets of kinases such as extracellular regulated protein kinases1/2 (ERK1/2), protein kinase B (AKT), p38, and c-Jun N-terminal kinase (JNK) which are specific to nicotinamide adenine dinucleotide phosphate (NADPH)-oxidase (Nox)-dependent and Nox-independent process, and subsequently lead to the decomposition of the membranes of granules and nucleus.^19^^,^^20^ In Nox-dependent formation, stimuli such as PMA, LPS, or fungi facilitate the assembly of Nox. Nox-independent formation can be activated through ionomycin, immune complexes, and platelets, involving calcium signaling and small conductance calcium-activated potassium channel protein 3 (SK3).^21^^,^^22^ These processes enable enzymes such as neutrophil elastase (NE), myeloperoxidase (MPO), and protein arginine deiminase 4 (PAD4) to enter the nucleus and decorate chromatin (Fig. 1).

## NETs and pulmonary infectious diseases

**Fig. 2:**
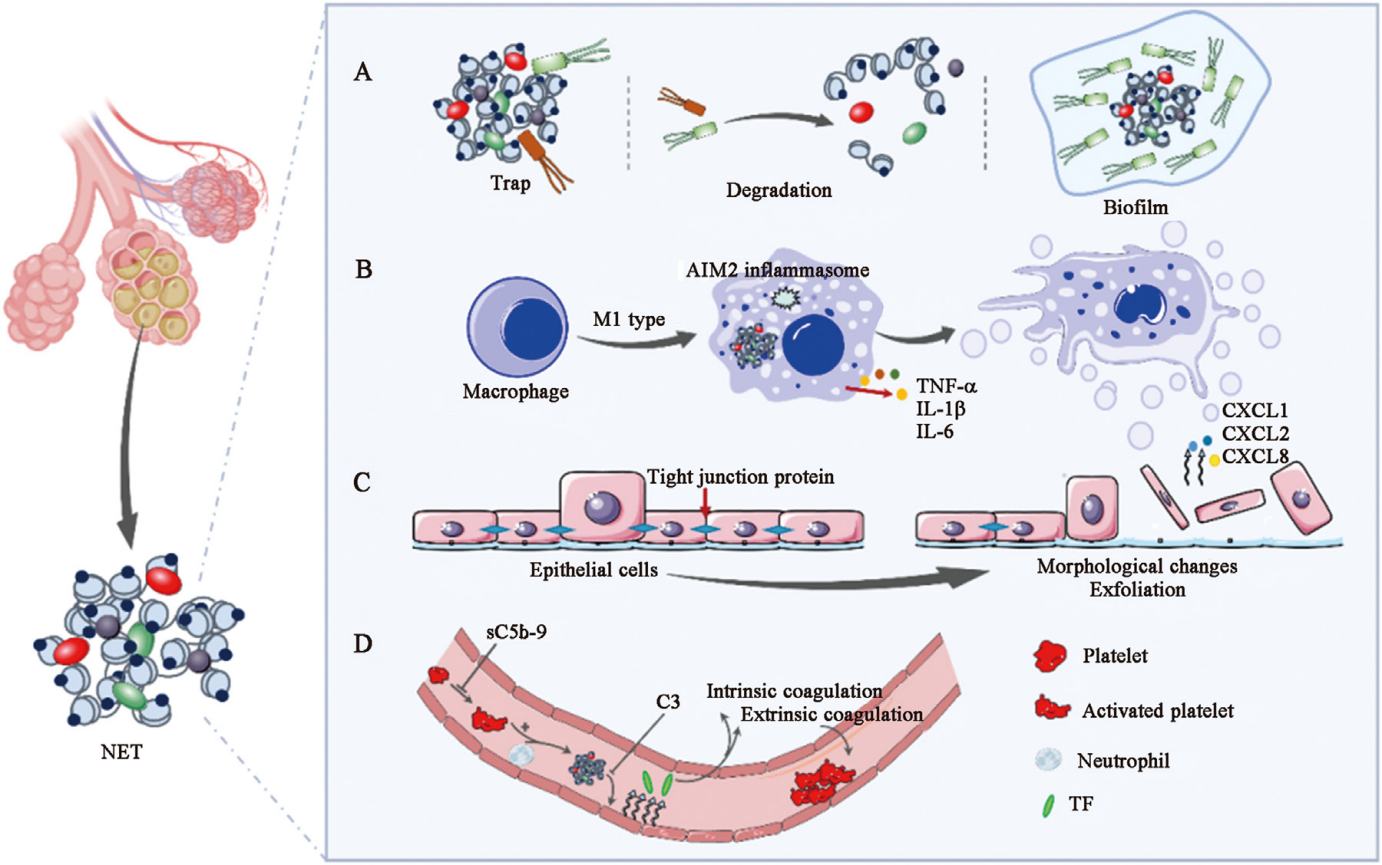
Mechanisms of NET-mediated pathology. (A) Three different models of bacterial interaction with NETs; (B) NETs aggravate the inflammatory response through macrophages; (C) NETs provoke epithelial injury; (D) NETs trigger immune thrombosis. AIM2: Absent in melanoma 2; C3: Complement 3; CXCL: C-X-C motif chemokine ligand; IL: Interleukin; NETs: Neutrophil extracellular traps; sC5b-9: Soluble phase-terminal complement complex; TF: Tissue factors; TNF-α: Tumor necrosis factor-α.

In contrast to the primary immune-protective function of NETs, the pathological damage caused by NETS has been further described. This section focuses on the pathophysiological role of NETs in pulmonary infectious diseases (Fig. 2).

### NETs and pneumonia

Pneumonia is among the most frequent cause of morbidity and mortality in the world.^23^^,^^24^ The most common bacterial pneumonia is community-acquired pneumonia (CAP), which is responsible for approximately 3.5 million deaths annually.^25^ The etiology of CAP is variable, and *Streptococcus pneumoniae, Haemophilus influenzae*, and *Klebsiella pneumoniae* are the most frequently identified causes of CAP.^26^

The innate immune system serves as the first line of defense against invading microbial pathogens. Once the bacterial infection is established in the lungs, neutrophils are massively recruited to the infection site and are responsible for eliminating microbes through three strategies: (1) phagocytosis of the microbe; (2) release of antimicrobial factors; and (3) formation of NETs to trap bacteria or viruses, which is followed by NET-mediated killing.^4^ Neutrophils are used to broadly combat bacterial infections by sensing the size of microorganisms and selectively releasing NETs, especially for large pathogens.^27^ A clinical case of a fatal non-typed *Haemophilus influenzae* (*H. influenzae*) infection with severe pneumonia and bacteremia has demonstrated a vast amount of NETs in the patient's sputum.^28^ Similar results have been obtained in diverse mouse models of bacterial pneumonia,^29^ suggesting an association between NET formation and severe respiratory infection. Anti-inflammatory and pro-inflammatory factors may have opposite effects on NETs. *Klebsiella pneumoniae* (*K. pneumoniae*) represents the major cause of hospital-associated pneumonia.^30^ Adenosine is a potent signaling molecule to inhibit inflammation, limit tissue injury, and promote repair.^31^^,^^32^ A 2012 study indicated that the deletion of adenosine A_2B_ receptor (A_2B_R) on bone marrow-derived cells resulted in an improved outcome of *K. pneumoniae* and at least in part, was attributable to enhanced NET production by A_2B_R-deficient neutrophils.^33^ Nevertheless, Mincle, as an inducible receptor expressed mainly by myeloid cells, triggers the FcRγ-Syk-Card9 pathway to induce the production of protective T-helper 1/T-helper 17 response, as well as chemokines required for the recruitment of inflammatory cells type.^34^, ^35^, ^36^ Mincle-deficient neutrophils display a defective ability to form NETs, leading to an increased bacterial burden and systemic dissemination.^37^ These data imply that although the ability to restrict inflammation and reduce tissue injury is beneficial, it inhibits the host response aimed against infections and might indirectly impair recovery in bacterial pneumonia. Moreover, type 2 diabetes (T2D) patients display elevated levels of NETosis but are vulnerable to carbapenem-resistant hypervirulent *K. pneumoniae* (CR-hvKP), due to deficient surface damage from NETs caused by T2D. Numerous pieces of evidence have indicated that some key antimicrobial components of NETs, including MPO and azurocidin (CAP37), are downregulated in diabetic patients with poor glycemic control,^38^ which could explain the increased susceptibility to CR-hvKP in T2D patients. Reduced formation and impaired function of NETs decrease bacterial clearance. Additionally, the increased NET formation can synergistically enhance the killing activity of macrophages by promoting phagocytosis and the transfer of neutrophil-specific antimicrobial peptides to macrophages, which has been demonstrated in response to *Staphylococcus aureus* and *Streptococcus pneumoniae* (*S. pneumoniae*) infection.^29^^,^^39^

Although the host immune system has multiple strategies for neutralizing pathogens, microbes have also developed various mechanisms to avoid being attacked.^40^ The filamentous NETs trap bacteria, making them vulnerable to phagocytosis and/or destruction by NET-bound antimicrobial proteins located in the NETs.^41^^,^^42^ A common evasion mechanism by microbes is to secrete enzymes that degrade NETs. For example, EndA, a surface endonuclease expressed by *S. pneumoniae*, can also degrade the DNA scaffold of NETs and destroy their functional integrity.^43^^,^^44^ Similarly, ArpA secreted by *Pseudomonas aeruginosa* (*P. aeruginosa*) can aggravate lung infection by effectively destroying NETs formation through degrading citrullinated histone H3 (Cit-H3) and MPO.^45^ Moreover, *P. aeruginosa* can utilize NETs as a scaffold for released biofilms, which allow the bacteria to hide in the matrix and be protected from the host immune defense, antibiotics, or chemotherapy.^46^ Ekaterina et al^47^ have confirmed that type I interferons (IFNs) lead to elevated NETosis, which in turn, triggers biofilm formation by *P. aeruginosa* and supports its persistence in the infected lung. The ubiquitous NET avoidance mechanism in respiratory pathogens suggests that NETs serve a critical antimicrobial role. Thus, future research should involve identifying additional mechanisms to diminish the effect of NETs by microbes (i.e., developing therapeutic interventions to counter these avoidance strategies).

Although the protective role of NETs in pulmonary infections has been clearly demonstrated in recent decades, many studies have suggested that excessive recruitment and activation of neutrophils are identified as a risk factor for acute lung injury (ALI)/acute respiratory distress syndrome (ARDS). This topic will be discussed in the next sections. Generally, maintaining the correct balance of NETs formation in the lung is critical for preserving the ability of NETs to contain microbes while minimizing damage to the host.^48^

### NETs and coronavirus disease 2019 (COVID-19)

Severe acute respiratory syndrome coronavirus 2 (SARS-CoV-2) has rapidly spread across the globe, with millions of people having been infected worldwide. COVID-19 resembles influenza, with a clinical picture ranging from mild upper airway symptoms to ARDS requiring ventilatory support.^49^ Decreased lymphocyte counts, reduced T-cell functionality and increased neutrophil-to-lymphocyte ratio are now well-established hallmarks of COVID-19.^50^, ^51^, ^52^, ^53^ Neutrophils are important effectors of damage in the lung and systemic circulation.^54^ Abundant NETs have been detected in the serum and lung tissue in COVID-19 patients, which have been described as major risk factors for mortality.^55^^,^^56^ *In vitro* experiments and clinical reports have indicated that SARS-CoV-2 can directly activate neutrophils to release NETs.^57^ Middleton et al^58^ have demonstrated that the ratio of partial pressure of oxygen in arterial blood (PaO_2_)/fraction of inspired oxygen (FiO_2_), a marker of severity of respiratory failure for which a lower score indicates increasing respiratory failure, varies inversely with plasma NET levels. Plasma NETs also correlate directly with the Sequential Organ Failure Assessment (SOFA) score, a clinical illness severity score. Plasma NET levels peak early after ICU admission and correlate with SARS-CoV-2 RNA load in the sputum and levels of neutrophil-recruiting chemokines and inflammatory markers in the plasma.^59^ Moreover, plasma NET levels decrease to levels similar to those of healthy adults when patients recover from COVID-19.^58^ These results suggest that NET can be used as a marker to assess disease progression.

NETs produced in COVID-19 patients also appear to trigger immune thrombosis, which has been verified in autopsy results.^60^ NET-induced intravascular coagulation requires the collaboration of numerous other components, mainly platelets, endothelial cells, clotting factors, and tissue factors (TF).^61^ Other factors are involved in thrombosis through the interaction with the platelet/NETs/TF/thrombin axis. When infected with SARS-CoV-2, platelets are activated with NET formation, followed by endothelial cell injury and blood vessel damage. Consequently, TF is exposed to the blood, causing the overexpression of clotting factors VII and VIII, activating the extrinsic or intrinsic coagulation pathways.^62^^,^^63^ Recent evidence has highlighted the function of complement in the before-mentioned process.^58^^,^^64^, ^65^, ^66^ Soluble terminal complement complex (sC5b-9) blockade attenuates platelet-mediated NET-driven thrombogenicity, and C3 inhibition can disrupt TF expression in neutrophils.^67^ Additionally, the ROS‑NET pathway also plays a role in thrombosis.^68^ Moreover, ROS can also promote venous thrombus formation through the modulation of the enzymatic cascade of fibrinolysis and the complement systems.^69^

Taken together, these findings suggest that the cytokine storm caused by SARS-CoV-2 is the trigger event of the pathological process in the lung tissue, which is followed by NETosis and endothelial activation and hemostatic imbalance, which ultimately determines clinical prognosis.

### NETs and ALI/ARDS

ARDS is a complex cascade process that develops from ALI, which is characterized by widespread inflammatory tissue injury.^70^^,^^71^ ARDS can be induced by various triggers, including infectious and non-infectious ones, such as pneumonia, sepsis, aspiration, non-cardiogenic shock, trauma, blood transfusion and inhalation injury.^72^

ARDS is used as an umbrella term to denote this inflammatory lung condition, but it is not clear whether it is one or multiple disease. An excessive inflammatory response is an important cause of increased mortality. Clinical and animal studies have demonstrated that higher expression of Cit-H3 and the formation of NETs in bronchoalveolar lavage fluid (BALF) than in the circulation suggests lung-borne production of NETs, suggesting that NETs might be associated with the onset and persistence of ARDS.^73^^,^^74^ In addition to infection, extrapulmonary factors, such as burn injury and chemical aspiration, can also cause severe lung injury by excessive NETosis.^23^^,^^25^^,^^26^ Phenylarsine oxide (PAO) causes a dermal chemical burn resulting in the activation of PAD4, which induces NETosis in a calcium- and endoplasmic reticulum (ER) stress-dependent manner. The direct detrimental effect of NETs in the lung tissue is to provoke epithelial injury. In response to NETs stimulation, epithelial cells undergo morphological changes and exfoliation with the degradation of tight junction proteins, such as occludin and claudin-1.^75^ Meanwhile, the toll-like receptor 4 (TLR4)/nuclear factor kappa-B (NF-κB) pathway is activated in the injured epithelial cells lines, thereby promoting transcription and translation of cytokines and chemokines, including C-X-C motif chemokine 1 (CXCL1), CXCL2, and CXCL8, which can cause the second wave of neutrophil recruitment. Therefore, NETs are responsible for triggering persistent lung inflammation. This may explain why some studies suggest a weak or non-effective effect of DNase I thearpy to eliminate NETs in lung injury since NETs degradation by-products and remaining proteinases can still stimulate epithelial cells to promote an inflammatory cascade. As described above, excessive NETs induce significant collateral damage to surrounding tissues and lead to platelet aggregation and microthrombus formation in the pulmonary vasculature, endothelial and epithelial cell death, loss of barrier function, and flooding of the interstitial space and alveoli.^32^^,^^48^^,^^76^^,^^77^

In addition to direct damage, NETosis can also cause functional disorders of the immune system. Recent research has focused on the relationship between neutrophils and macrophages. During ARDS, the lifespan of neutrophils is prolonged, and apoptosis is delayed.^78^, ^79^, ^80^ Excess neutrophils undergo NETosis to produce a large number of NETs. On the one hand, NETs promote macrophage transition to pro-inflammatory phenotype (M1 type).^81^^,^^82^ On the other hand, macrophages, which engulf NETs, lead to absent in melanoma 2 (AIM2) inflammasome activation and pyroptosis, followed by the release of robust cytokines. In turn, pyroptosis boosts NET levels in BALF by promoting neutrophil influx into alveoli.^83^ In addition, the thick sticky secretions, containing eDNA and protein are more likely to cause airway obstruction, which can worsen patient breathing.^12^

NET is an amplifier of the inflammatory response, which can exacerbate the degree of tissue damage through this vicious cycle. Tissue repair and host recovery must be started, which requires the inhibition of inflammation to alleviate ARDS. Therefore, targeted inhibition of NET formation or accelerated degradation is a promising therapeutic approach.

### NETs and cystic fibrosis (CF)

CF is a lung disease characterized by chronic inflammation of the airways associated with bacterial colonization. It is caused by mutations in the CF transmembrane conductance regulator (CFTR), which encodes a transmembrane anion channel transporting chloride and bicarbonate. Neutrophils account for 80% of the total cells present in CF sputum.^84^ Hypochlorite is a potent ROS found within the phagolysosome and responsible for microbial digestion.^85^ CFTR^−/−^ neutrophils have impaired the transport of chloride from the cytoplasm into the phagosomes, leading to an increased cytosolic concentration of Na^+^ and Cl^−^.^86^ The abnormal pH of CFTR^−/−^ neutrophil cytoplasm increases the release of antimicrobial enzymes such as MPO and NE^87^ and accordingly overwhelms the binding capacity of the anti-protease α1-antitrypsin which eventually contributes to lung destruction and bronchiectasis.^85^^,^^88^ Atomic force microscopy (AFM) and scanning electron microscopy have been employed to characterize the nature of CF sputum and have found that CF sputum is predominantly composed of a high-density meshwork of NETs and NETosis-derived material.^89^ Compositional breakdown analysis of sputum proteomes in CF patients has found S100-A9, the histone H2B family, lactotransferrin, histone H4, cathepsin G, and MPO are consistently present in high abundance, all of which are constituents of NETs.^90^ The clinical association between higher DNA levels in airway fluid and worsening lung function in CF patients illustrates the adverse effects of NETosis on lung inflammation and disease progression.^91^

Infection is also an important feature of CF. The microbiotas in CF airways are diverse, including *P. aeruginosa* and *Burkholderia ceponiae*.^92^^,^^93^ Other bacterial groups that have been found in the airways of CF patients include *H. influenzae, Stenotrophomonas maltophilia, Colorless Bacilli xanax, Pandoraea,* and *Streptococcus*.^94^^,^^95^ The paradox of CF lung disease is that plenty of neutrophils are present in the airway lumen, but they are not effective in controlling bacterial infections. Dwyer et al^96^ have carried out experiments using the non-mucoid strain PAO1 and the mucoid clinical isolate PA2192 to model the survivability of early (non-mucoid) *vs.* late (mucoid) colonizers. Their data suggested that only 20% of mucoid strain PA2192 was trapped but not killed by NETs.^96^ In another study, pyocyanin, as the most important *Pseudomonas* virulence factor, has been able to promote NET formation in a time- and dose-dependent manner with the involvement of NADPH oxidase. Moreover, a large amount of free DNA accumulation promotes bacterial colonization and biofilm formation and increases mucus viscosity.^13^^,^^14^^,^^97^ Therefore, NETosis in CF airways eventually fails to eradicate bacterial infection resulting in increased pathogenicity of the bacteria. The long-lived NET structures provide a niche for infection establishment and persist at the cost of harm in patient's health.

The pathogenic role of NETosis in CF has been expanded to include the development of autoimmunity. Bactericidal/permeability-increasing protein (BPI) is a potent antimicrobial protein stored in neutrophil azurophilic granules, with bactericidal properties specific for Gram-negative bacteria (GNB). CF patients develop anti-BPI autoimmunity, levels of which correlate with diminished lung function.^98^, ^99^, ^100^ A recent study has proposed that an increase in autoreactivity to BPI was a result of *P. aeruginosa-*mediated NETosis, leading to BPI cleavage and production of a 30 kD C-terminal BPI fragment.^98^ *S. aureus*-induced NETs are thought to manifest as autoantibody targeting proteinase 3 (PR3) and pathogenic *S. aureus* protein.^101^^,^^102^ In a clinical trial, serum NET-associated autoantibodies have been analyzed from the following number of subjects: 37 CF patients, 23 healthy controls (HC), 20 rheumatoid arthritis (RA) patients, and 21 systemic lupus erythematosus (SLE) patients. Levels of anti-PAD4 antibodies were elevated in CF patients compared to HC and showed an inverse correlation with a measure of lung function, namely forced expiratory volume in one second (FEV_1_)% predicted and MPO levels. Notably, the elevation was associated with *P. aeruginosa* but not with *S. aureus*.^103^ These unique autoantigen signatures suggest that not all NETs are equal and that specific environmental or chemical stimuli determine their disease specificity, which should be taken into account in the future when investigating how antigen-specificity is generated and new targeted drugs or evaluation indicators are developed.

## NETs as a therapeutic target

Generally speaking, despite the ability to fight invading microorganisms, excessive NETs can play a role in disease progression, where they provide a stimulus or scaffold for cytokine storm, thrombosis, tissue damage, and tumor metastasis. Therefore, it is critical to avoid disrupting the protective effects of NET formation and the release of potentially destructive byproducts of NET degradation in developing new therapeutic interventions targeting NETs.

In the early stage of infection, NETs capture the bacteria, thereby reducing their spread by confining them to specific areas. Especially for susceptible persons with underlying diseases, reduced NETs formation and impaired function decrease bacterial clearance. Intentional promotion of NETs is prone to lead to uncontrollable inflammation, eventually leading to ALI or ARDS, while the inhibition of bacterial escape mechanisms can synergistically increase the effectiveness of NETs.^104^ For patients with chronic infections such as CF or tuberculosis, the long-living NET structures provide a niche to establish the infection that persists at the cost to patients’ health. Thus, the choice of NET-targeted therapy should depend on stages and pathogens in the disease course. Certain types of pathogens, cytokines, and airway surface liquid (ASL) pH have been reported to influence the bactericidal activity of neutrophils and NETosis.^105^ Therefore, a further understanding of the molecular mechanisms of NETosis (including the effects of reduced pH, metabolic pathways, kinases, sodium/potassium channels, and transcription regulation) would contribute to identifying potential therapeutic targets to control excess NETosis without altering the antimicrobial functions.

Recombinant DNase I is a common drug that can efficiently break down the chromatin within NETs, which contributes to immune-mediated thrombosis and luminal obstructions of airways and vessels.^106^ Several clinical trials studying the therapeutic effect of DNase I have been approved since the COVID-19 outbreak,^107^^,^^108^ and various dosage forms and delivery methods, such as atomization inhalation or nanomaterials, have been developed.^55^^,^^109^ Additionally, IL-8, as one of the powerful NET stimuli, is another promising target, as demonstrated in some pre-clinical research.^110^^,^^111^ In animal models of ALI, the inhibition of high-mobility group box 1 (HMGB1), which has been shown to promote NETs release,^112^ alleviates sepsis-induced ARDS.^113^ Moreover, the downregulation of HMGB1 can also increase the ability of macrophages to clear NETs and neutrophils.^114^ As described above, NETosis is a node connecting multiple pathological processes. Therefore, many drugs or therapies that regulate immune response, inhibit neutrophil aggregation, and maintain the balance of coagulation function can affect NET production, ultimately alleviating symptoms and improving prognosis. For instance, many studies have confirmed the beneficial effects of Re-Du-Ning,^115^ mesenchymal stem cells (MSCs),^116^^,^^117^ heparin,^106^^,^^118^ erythromycin,^119^ and other inhibitors associated with inflammatory response.^120^^,^^121^ Moreover, increase in mucus viscosity and mucus plugging of small and medium-sized bronchioles impede the migration of monocytes to the alveoli. Therefore, reducing mucus viscosity and mucus plugging of small and medium-size bronchioles may improve the migration of monocytes to the alveoli, thus affecting the phagocytic clearance of NETs by macrophages. Hence, not all therapies are necessarily specific. However, it is difficult to eliminate the persistent effect of NETosis, especially with monotherapy. Moreover, although NETs are a common feature of most lung diseases, they manifest different characteristics in different diseases from a pathological point of view. Thus, identifying highly specific targets for NET generation and clearance is crucial for the development of therapies.

## NETs and prognosis

In various pulmonary diseases, especially in the acute phase, NET formation tends to be positively correlated with the severity of the disease and changes dynamically over the clinical course. A greater burden of NETosis has been reported in patients who require mechanical ventilation than in those not requiring oxygen supplementation.^122^ Distinct from apoptosis, toxic neutrophil contents are no longer retained inside but released into the extracellular space, which constitutes a mechanism of hyperinflammation, amplifies disease severity, and leads to death.^123^ Moreover, residual irritation and unrepaired tissue damage perpetuate NETs even after remission in the acute phase, thus promoting the eventual chronic state. For chronic lung infectious diseases such as CF, NETosis in the airway eventually fails to eradicate the bacterial infection, but instead increases pathogenicity, leading to impaired lung function and extensive airway obstruction, which reduces the quality of life and increases patients morbidity. The formation of NETs in the pathogenesis of lung disease poses a difficult challenge for the development of therapeutics.

The close relationship between NETs and pulmonary disease makes measuring levels of NETs as a potential biomarker promising in clinical use. For example, according to a secondary analysis of a randomized, controlled, multicenter trial, NETs have been significantly associated with increased morbidity in patients with CAP, while prednisone treatment has modified circulatory NET levels with beneficial outcomes.^124^ The potential and value of NETs for therapeutic monitoring and prognostic prediction have also been demonstrated in several prospective studies on COVID-19.^58^^,^^125^ Since the pulmonary microenvironment varies in different diseases,^38^ NETosis, as the most common pathologic process, has great potential in disease surveillance, treatment evaluation, and prognostic prediction when linked with clinical indicators.

## Conclusion

NETs have been originally identified to have a crucial role in the host defense against microorganisms. However, the long-term presence of NETs is linked with host tissue injury and risk for the development of autoimmunity. As a novel link between innate and adaptive immune systems, the formation and activation of NETs can partly explain why chronic inflammation persists even when acute exacerbation is relieved. An excess or persistence of NET release is potentially injurious to host organs and cells, leading to exacerbation and perpetuation of many diseases. Thus, maintaining a critical balance of NETs is necessary, which has important therapeutic implications for preventing lung injury and maintaining microbial control. In view of the complex physiological and pathological structure of the lungs, the prognosis is difficult to predict. Therefore, the timing to promote or shut down NETs will be a challenge in future research.

## Funding

This present review was supported by the National Natural Science Foundation of China (Nos. 82130001, 82070045) and the National key Research & Development plan (No. 2020YFC2003700).

## Conflicts of interest

The authors declared that they had no competing interests.
